# Collectanea: Miscellaneous

**Published:** 1835-07-01

**Authors:** 


### MISCELLANEOUS.

### IMAGINARY NEW EDITIONS.

An honest reviewer is sometimes tempted to say of the book he is noticing, "This is *really* a new edition;" a phrase which we will explain in all its bearings for the benefit of innocent country readers, who must be as much surprised when they hear of spurious second editions, as when they are told of our porter medicated with morphia and strychnine, or our bread rich in sulphate of alumina and phosphate of lime.

Second editions may be conveniently divided into three classes.

First, in the genuine second edition the sheets of the former one are not made use of; the types having been distributed, are again set up; and the author, making use of his opportunities, not only corrects false prints, bad grammar, misty sentences and loose theories, but endeavours in every page to raise his book to a level with the knowledge of his time. This is the second edition of the golden age.

Secondly, we have the *quasi* second edition. In this product of human frailty the sheets of the former impression are used, but a supplement is added; or perhaps a few leaves are cancelled, and new ones inserted in order to correct some very glaring errors. Such an edition belongs to the silver age; it is unsatisfactory to the reader who hopes, but in vain, that the information contained in the book will be brought up to the year, which is printed on the title-page.

Thirdly, we see the spurious second edition, which differs from the first in its title-page, and perhaps in its preface, but is otherwise the same; thanks to the superabundant chlorine of the paper-bleachers, it is often betrayed by its chlorotic complexion, for our modern paper loses its colour, or rather gains one, in the space of a few years. This knavish attempt to save good for nothing books from their natural destiny belongs to the present time, the age of iron, or of brass.

These things being so, it is passing strange that some critics should say that the words fourth edition or fifth edition on the title-page save the necessity of examination, and speak volumes for the book. Are they unable to detect the most flagrant impositions, or, in the spirit of Pope's universal prayer, do they hide the fault they see? Would it not be still more strange if a writer on any branch of physic, in order to excuse the identity of his editions, were to tell us that ten or more years had passed away since his last edition, but that he had no improvements to make?

> Who would not laugh, if such a man there be,
> Who would not weep, if Atticus were he?

The detection of these pseudo-second editions is easily effected by a typographical test. If we find that the errata of the first edition, even those of the most glaring kind, occur in the second, each constant to its place, we may be sure that this is not the effect of accident: when the throws can be named beforehand, the dice are loaded.

This assumption of honours in the republic of letters by puny whipsters, who have no claim to them seems to us a weighty misdemeanour, and one which should be punished by public exposure in several conspicuous reviews. ARISTARCHUS.

### THE RAPIDITY OF THOUGHT.

All popular and settled notions, however unfounded, like prejudices early imbibed, are with difficulty eradicated. Among these may be instanced the dictum of the astonishing rapidity of Thought, which is almost proverbial, and generally believed: even Mr. Tooke, Vol. I., p. 28, conforms to this established maxim. "Words have been called *winged:* and they well deserve that name, when their abbreviations are compared with the progress which speech could make without these inventions; but when compared with the *rapidity of thought*, they have not the smallest claim to that title." By calculation, the progress of light from the sun and other luminaries is said to be ascertained; and likewise the rate at which sound travels: but hitherto no contrivance has been fabricated to estimate the rapidity of thought. If the succession of our thoughts should be more rapid than they can be distinctly apprehended, confusion must ensue, and their rapidity would render them useless. Our preceptions are regulated by the same law. If the prismatic colours be painted on a surface which is revolved with great rapidity, the individual colours will not be apparent. The succession of sounds to a definite number, may be severally distinguished, in a certain interval: but if the succession be increased beyond the power of discrimination, they will impress the ear as one uniform sound. The same principle must regulate our thoughts, whether they be composed of Ideas or words, or, if it be possible, of both jumbled together. It does not appear that our thoughts for any useful purpose, which must imply their communication to others, or for a record in written characters, *can* be more rapid than the intelligible pronunciation of the words themselves, and which, when delivered in quick succession, leave the shorthand-writer behind.*

*Dr. Haslam on Thought.*

[We side with the people, and Horne Tooke. It is obvious that besides the two useful purposes of thought allowed by the author, there is a third, of which almost every one is conscious. This is effected when the mind is employed in skimming over familiar ideas to arrive at some new master-thought. Excited by unexpected combinations, or soothed into a delicious reverie, the happy thinker could no more give an account in writing of each idea, than one who gazes on a stream could give an account of each particle of water that flows before him. *Ed. Med. Quart.*]

### INQUEST AT DUBLIN.

*An accurate Report of a curious Inquest and unparalled Case which occurred on Wednesday, the* 1*st of April*, 1835. Such is the title of a pamphlet of fifteen pages published at Dublin, the substance of which may be given in a few words. Mr. Hayden, a surgeon, having been called in to a case of lingering labour found it necessary to open the head of the child; a coroner's inquest was held upon the body, and the following verdict returned:

* It is very probable that Martial, in his eulogy of the Roman Notarius may have exceeded the actual performance.

"Currant verba licet, manus est velocior illis:
Nondum lingua suum, dextra peregit opus." *Lib.* 14, *Epig.* 208.

"We find, that the Child, in relation to which our inquiry has been held, was in all probability dead before the operation was performed by Surgeon Hayden; and we further find, that even if the fact were otherwise, then, that the operation performed by Surgeon Hayden, was both well-judged and well-executed, and the result of which has been the saving of the life of the mother: which would, as it appeared on evidence, have been sacrificed by the delay or omission of such operation—and we feel it our bounden duty to give expression to these sentiments, lest any, the slightest, imputation might be thoughtlessly attached to Surgeon Hayden." P. 15.

Mr. Hayden is naturally very angry that an inquest was held at all, and attributes it to the spleen of a Mr. Buckley, an apothecary, who was mortified that the medicines sold at the Bishop-street hospital were preferred to his. There certainly appears to have been no reason for the inquest, but we could wish that craniotomy was never performed without a consultation.

### DISEASES AND WOUNDS OF PLANTS.

Plants, perhaps, suffer more from invermination and the attacks of insects than from any other means; yet they are subject to other diseases, both of a sporadic and epidemic kind. Some of these even bear a similitude to animal disorders, and have, therefore, received similar names, of which Wildenow furnishes a catalogue. Thus, plants are affected with atrophy, tabes or consumption, anasarca or dropsy, hæmorrhage, lepra, verrucæ, or warts, chlorosis, icterus, ulcerations, common gangrene, and necrosis, or dry gangrene, besides various kinds of deformities, wounds, mutilations, &c. &c. They are likewise subject, especially the cacti, to a peculiar kind of sudden death, called by the French "la mort," by which, when affected, a branch or even a whole plant is as rapidly destroyed as the use of a limb is lost, or death produced in animals by apoplexy.

Plants, although they will bear judicious pruning, resent barbarous operations, and even accidental injuries. In them, as in animals, contused wounds, especially contused and punctured wounds, are much more dangerous than incised ones; of this there is an example in the adjoining green-house. It is a splendid agave or American aloe; this, which is one of its leaves, was pierced last season with the ferrule of an umbrella by one of the visitors to the garden. The parenchymatous substance became diseased; it sphacelated, and the mortification, which at first extended upwards, subsequently began to descend and travel so rapidly towards the base as to render amputation necessary. The operation was performed, but, as you will perceive on examining the plant, the mortification had previously extended in an insidious manner so far towards the centre that a fatal termination is to be feared.—*Introductory Lecture delivered in Chelsea Garden, on Monday, April 27th,* 1835, by Professor Burnett.

